# Treatment of recalcitrant femur nonunion with pedicled corticoperiosteal medial femoral condyle flap

**DOI:** 10.1038/s41598-023-47432-x

**Published:** 2023-11-21

**Authors:** Ali Özdemir, Egemen Odabaşı, Ebubekir Eravsar, Selim Safalı, Mehmet Ali Acar

**Affiliations:** 1 Department of Orthopaedics and Traumatology and Hand Surgery Konya, Selcuk University, Konya, Turkey; 2 Department of Orthopaedics and Traumatology, Konya Beyhekim Training and Research Hospital, Konya, Turkey; 3 Department of Orthopaedics and Traumatology, Konya City Hospital, Konya, Turkey

**Keywords:** Medical research, Trauma

## Abstract

Periosteal or osteoperiosteal medial femoral condyle (MFC) flaps may be good options for atrophic nonunion. The aim of this study was to evaluate the effectiveness and safety of pedicled MFC flap in the treatment of recalcitrant femur nonunion without bone defect. Thirteen patients (11 male and 2 female), who suffered recalcitrant femur nonunion and were treated with pedicled osteoperiosteal MFC flap between January 2014 and April 2018, were included in this study. Patient files were reviewed retrospectively. Atrophic or recalcitrant 2/3 distal femoral nonunion were the indications for this clinical procedure. Demographics and operative data, flap size, visual analog scale (VAS) score, time to union, and complications were evaluated. A total of 13 patients underwent femur nonunion treatment with MFC flap after an average of 3.4 previous surgical procedures. The median age was 34 (Q1: 32.5, Q3:43) years old. The mean flap size was 4.3 × 6.4 cm, all nonunions healed in a median 5 months (Q1: 4.5, Q3: 6). There were an intraoperative knee medial collateral ligament injury in a patient, hematoma in a patient, and seroma in two patients. The median length of the follow-up was 40 months (Q1: 30, Q3: 47). There wasn’t any additional complication in long-term follow-up. Functional outcomes were satisfactory. The median preoperative VAS score was 7 (Q1: 6, Q3: 9.5), decreasing to 1 (Q1: 0, Q3: 1) at the 6-month follow-up, and further reducing to 0 (Q1: 0, Q3: 1) at the 24-month follow-up. The nonunion period ranged from 6 to 18 months. The pedicled MFC flap is a good option for recalcitrant femur nonunion where larger vascularized flaps are not warranted. It is easy to harvest, does not require microvascular anastomosis, is effective, and offers minimal donor site morbidity.

## Introduction

Although nonunion occurs in approximately 5–10% of fractures^1^, fracture healing is a unique and well-organized process with stability and local biology. Autogenous bone graft is the most common option for nonunions and union rates are satisfactory following plate fixation with the iliac crest bone graft ^2^. However, approximately 20% of fixation revision and bone grafting procedures may result in failure^2^. Variable outcomes and complications have been reported in nonunions with many treatment options, such as exchange nailing, dynamization, external fixation, stem cell injection, and dynamic compression plate. For recalcitrant nonunions, treatment is a challenge for both the patient and the surgeon due to lack of vascularity and deficits of the bone’s biological potential. Unfortunately, the success rate of re-applied non-vascularized bone grafting is limited due to the deterioration of soft tissue support secondary to recurrent surgical interventions, previous infections, and impaired bone vascularity at the fracture site^3,4^. For such situations, bone flap transfers should be considered to achieve a favorable bone union.

Bone flap protects osteocytes and accelerates graft consolidation without the need for creeping substitution^5^. A free fibular flap is a gold standard option in wide bone defects, but it is not appropriate for smaller defects (< 3 cm), and the iliac crest flap can be considered as an alternative. Sakai et al. introduced free medial femoral condyle (MFC) periosteal flap in the management of recalcitrant nonunions without bone defect^6^. Periostal flaps are more flexible and have excellent osteogenic capacity since the cambium layer is preserved^7^. MFC periosteal flap is generally used as a free flap in the upper and lower extremity nonunions^8,9^. Although studies demonstrating the use of the pedicle type of this flap in femur nonunions are scarce ^12–15^, we envisioned that it would become increasingly important in the treatment of persistent femoral nonunion owing to its high osteogenic capacity.

Herein, we aimed to present the clinical and radiological evidence of 13 patients who were treated with pedicled MFC flaps to manage the difficult-to-treat persistent nonunion of the femur.

## Methods

Thirteen patients, who were treated with pedicled periosteal MFC flap between January 2014 and April 2018, were retrospectively evaluated. The study was approved by the local ethics committee and was conducted in accordance with the ethical standards of the 1975 Declaration of Helsinki revised in 2008. The inclusion criteria are as follows: recalcitrant atrophic nonunion of the distal two-thirds of the femur treated with pedicled periosteal MFC flap, at least 6 months after the last fracture treatment. Nonunion was ascertained through clinical and radiological evaluations. Clinically, it was determined by the manifestation of pain or the inability to bear full weight. Radiographically, nonunion was delineated by a fracture exhibiting no indications of healing for a minimum of three months or lacking radiographic evidence of union for at least nine months^10^. We excluded certain patient groups from flap application, specifically those under 18 years of age and individuals with infected nonunion. The demographics and clinical characteristics of the patients, including age, sex, and previous operations were obtained from the hospital medical archive and patients’ anamnesis. Distance of nonunion to distal femoral condyle, operation time, time to get union, complications, functional results, and size of the flap were assessed. Radiographs were conducted monthly until fracture union. Subsequently, the radiographs obtained at the last three visits were collected and analyzed. The bridging of three cortices on two viewed radiographs was considered bone union. Functional assessments were conducted with each patient’s level of pain feedback using the visual analog scale (VAS), where 0 indicates the absence of pain and 10 indicates the maximum pain.

### Statistical analysis

Continuous variables were presented as median and quartiles. The normality distribution of continuous data was assessed through the Shapiro–Wilk test, hologram, skewness, and kurtosis values. In comparisons employed the related samples Wilcoxon signed rank test for non-normally distributed data and paired sample t-test for normally distributed data. Statistical significance was set at *P* < 0.05.

### Surgical technique

#### Flap harvesting

**Figure 1:**
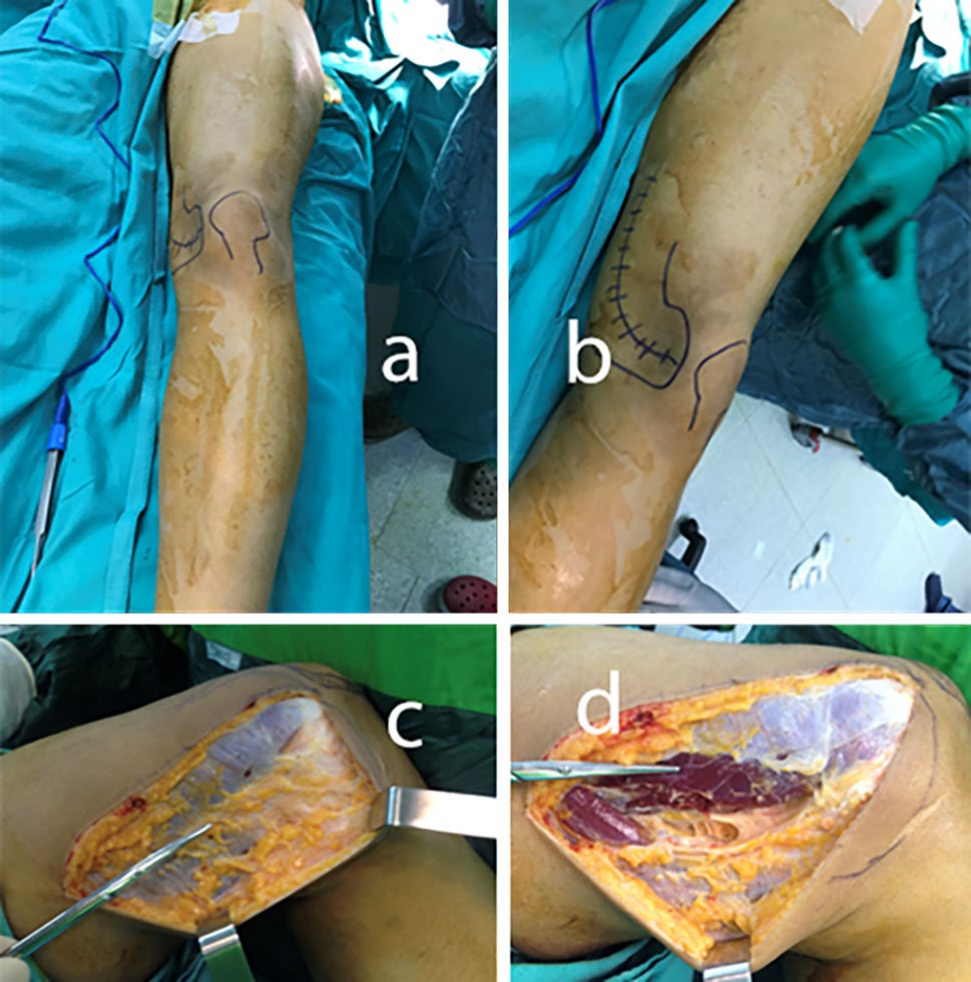
Preoperative surgical incision drawing (**a** and **b**), fascia of vastus medialis (**c**) and longitudinal incision of fascia (**d**).

**Figure 2:**
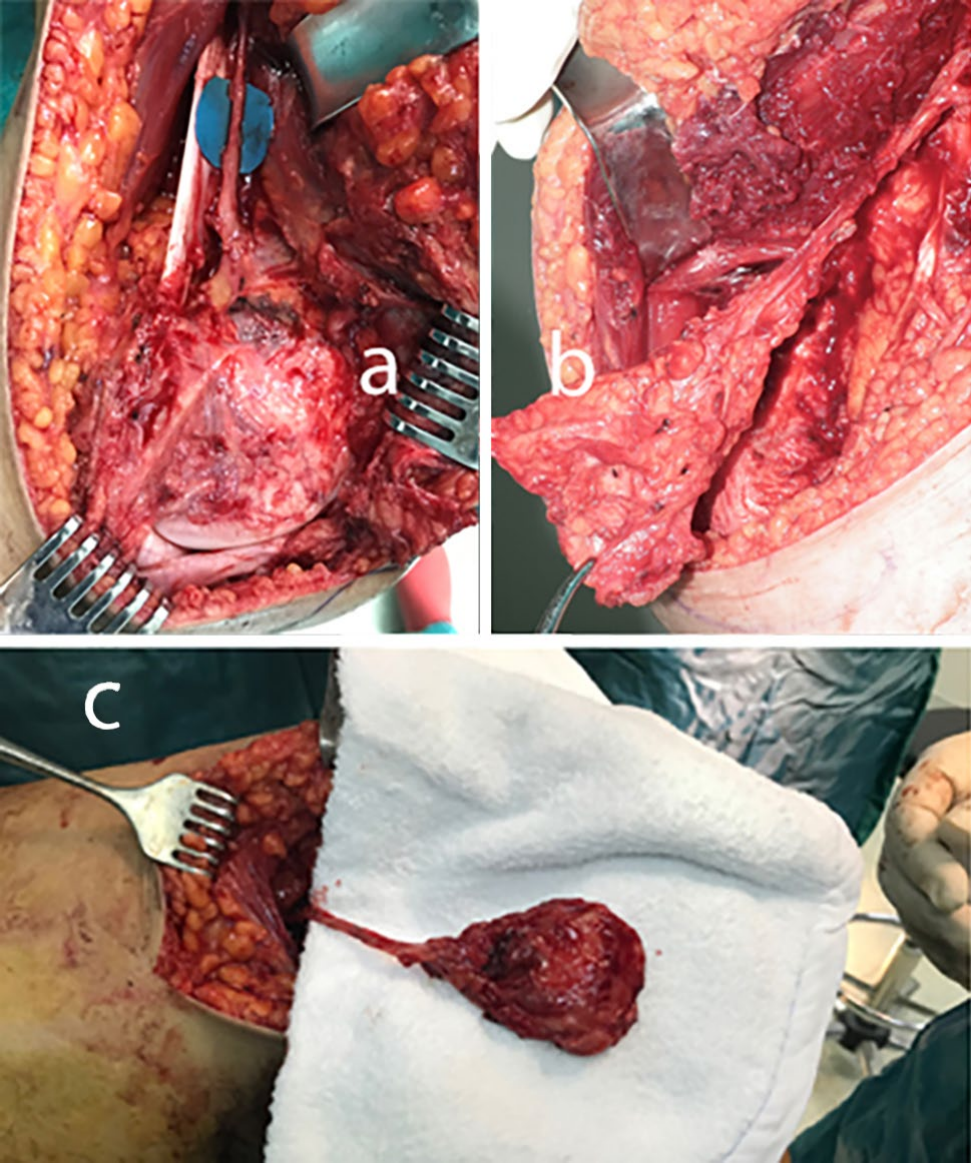
Dissected DGA and flap pedicle (**a**), harvested MFC flap (**b**) and pedicled MFC osteoperiosteal flap (**c**).

The same surgeon operated on all patients. Flap anatomy, as well as harvest technique, has been previously described by Sakai et al.^6^ and surgical procedures were performed as previously described. First, the patients are placed in supine position (Fig. 1a and b). The incision is made from the medial aspect of the patella along the posterior musculus vastus medialis (Fig. 1c). The fascia of the vastus medialis muscle is retracted anteriorly (Fig. 1d). DGA is determined (Fig. 2a). At this stage, posterior retraction of the Sartorius may be required in order to facilitate dissection of the artery. The medial superior genicular artery is identified and ligated. Then, dissection is continued until the MFC longitudinal and transverse branches are exposed. The flap harvest proceeds from the distal to the proximal to prevent injuries to the vessels. The periosteum is elevated circumferentially by using osteotomy if it is planned corticoperiosteally, or with a periosteal elevator if it is planned periosteally. The flap boundaries include the medial patellar facet as the anterior margin, the posterior border of the femur as the posterior margin, and the origin of the medial collateral ligament (MCL) as the distal margin. The surface of the knee joint and MCL is preserved during flap preparation. The DGA is dissected proximally, where it originates from the adductor hiatus (Fig. 2b and c).

#### Preparation of the nonunion site

By extending the medial incision, debridement is performed on the nonunion area. Then, the pedicled MFC periosteal flap is wrapped around the nonunion site without stretching and fixed with sutures. The corticoperiosteal flaps are not sufficient to totally cover the femur. Thus, it is wrapped to cover the nonunion site as much as it can and then fixed with a suture.

### Postoperative care

Enoxaparin sodium treatment was maintained for a minimum period of 4 weeks. Knee/hip mobilization and quadriceps exercises were ordered after the 2nd postoperative day. Patients were discharged on the 3rd postoperative day.

### Ethical approval

This study was carried out after the approval of the local instutional review board (Selçuk Univercity Medicine Faculty Ethical Committe).

### Informed consent

Written informed consent was obtained from all subjects and/or their legal guardian(s). The local ethics committee of Selcuk University Medicine Faculty approved informed consent.

## Results

Thirteen patients (11 men and 2 women), who had recalcitrant nonunion of the distal two-third of femur, were included in the study. The median age was 34 (Q1: 32.5, Q3:43) years. The etiologies of the fractures were vehicle accidents in 10 patients, falls in two patients, and gunshot injury in one patient. Six patients had isolated femoral fractures, whereas seven patients had additional fractures. 7 of 13 patients had smoking history. Any patient presented with infection in postoperative follow-up. The nonunion period before the flap surgery ranged from 6 to 18 months (median 11, Q1:9-Q3:16). All patients had an average of three previous reconstructive attempts for their nonunions (range: 3–5), including the exchange retrograde intramedullary (RIM) nail, nail dynamization, iliac crest bone grafting, allograft, artificial growth factors (bone marrow injection, autologous platelet-rich plasma ), and bone stimulator (Table 1).

**Table 1:** Patients demographics.

Case no	Gender	Age	Location of nonunion	Smoking	Comorbidites	Previous surgical attemps	Nonunion time from the last surgery (months)	Follow up (months)
1	M	50	M 1/3	Yes	T2 DM	3	9	46
2	M	33	M 1/3	No	None	4	6	48
3	M	36	M 1/3	Yes	None	3	11	72
4	M	34	M 1/3	Yes	None	3	16	60
5	M	34	D 1/3	No	None	3	8	42
6	M	32	M 1/3	Yes	None	5	9	28
7	M	34	M 1/3	Yes	None	4	18	24
8	M	35	M 1/3	Yes	None	3	12	42
9	M	20	M 1/3	Yes	None	3	16	28
10	F	65	M 1/3	No	T2 DM	4	9	36
11	M	36	M 1/3	Yes	None	3	18	40
12	M	27	M 1/3	Yes	None	5	12	38
13	F	65	M 1/3	No	Hypertension	3	9	32

*M* male, *F* female, *M 1/3* Medial 1/3 of femur, *D1/3* Distal 1/3 of femur, *T2 DM* Type 2 Diabetus Mellitus.

**Figure 3:**
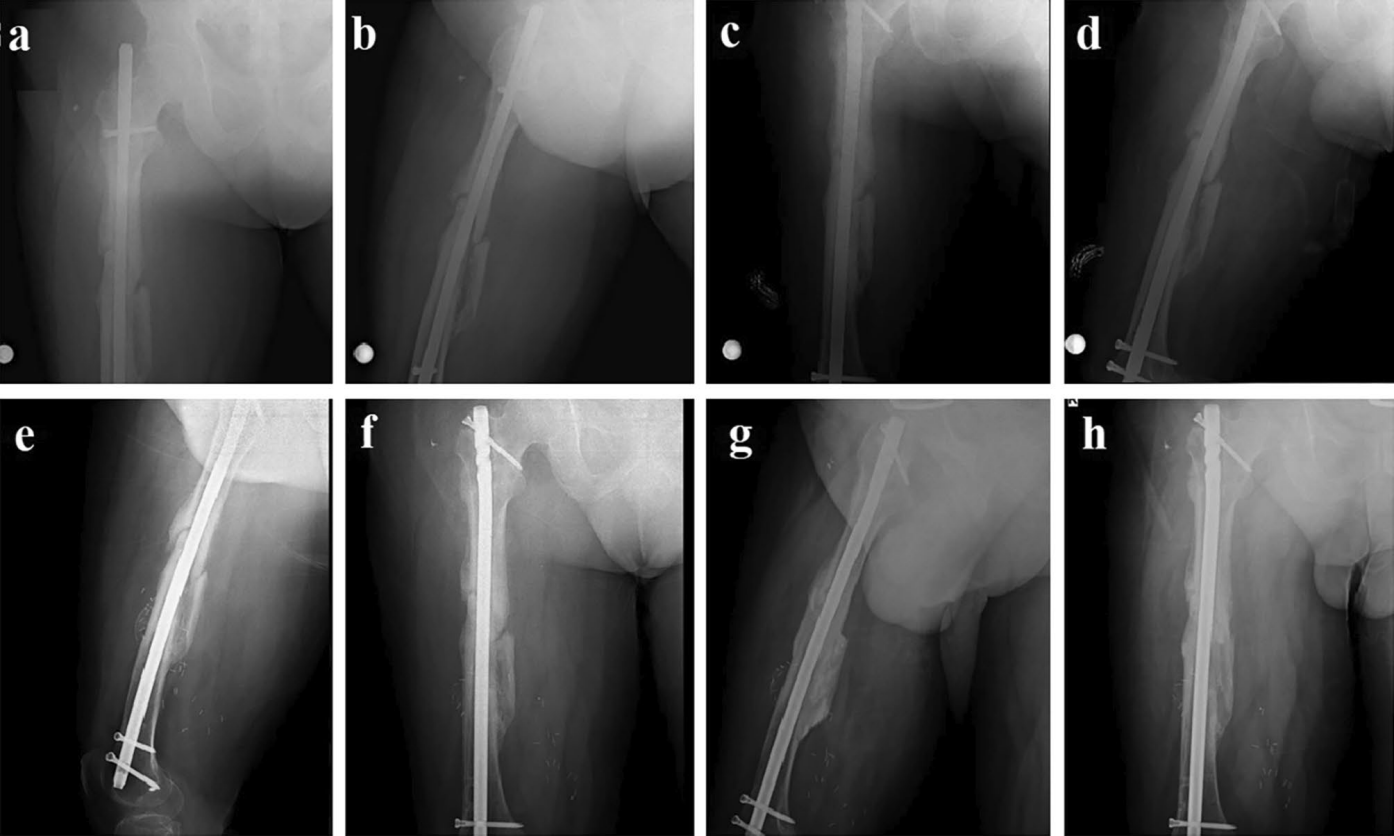
Preoperative nonunion radiographs (**a** and **b**), first step treatment: Exchange nailing (**c** and **d**), pedicled osteoperiosteal flap surgery (7 months later after exchange nailing) (**e** and **f**), postoperative 6th months union radiographs (6 months later after MFC flap) (**c** and **d**).

The median surgical time was 80 min (Q1:70, Q3:90), and the median time for flap harvesting was 60 min (Q1:50, Q3:67.5). The median pedicle length was 11 cm (Q1:10, Q2:12). The mean flap size was 4,3 × 6.4 cm. The median length of the follow-up period was 40 months (Q1: 30, Q3: 47). The median healing time for all nonunions was 5 months (Q1: 4.5, Q3: 6). (Fig. 3). Radiological bone union was evidenced by the presence of juxtacortical bony bridging as early as 4 to 8 weeks. The median preoperative VAS score was 7 (Q1: 6, Q3: 9.5), decreasing to 1 (Q1: 0, Q3: 1) at the 6-month follow-up, and further reducing to 0 (Q1: 0, Q3: 1) at the 24-month follow-up. Statistical analysis revealed a significant difference regarding preoperative and postoperative VAS evaluation (p < 0.5) both at 6 and 24 months follow-up (Table 2).

**Table 2:** Intraoperative findings and postoperative results.

Case no	Pedicle lenght (cm)	Distance between condyl to fracture (cm)	Complications	Time to union (months)	VAS preop	VAS postop 6. months	VAS postop 24. months
1	11	19	MCL injury	6	6	1	1
2	12	14	None	3	10	1	1
3	10	20	None	5	7	1	0
4	12	16	Hematom	6	7	0	0
5	12	13	None	5	5	1	1
6	9	15	Seroma	4	9	4	3
7	11	13	None	5	6	0	0
8	13	15	None	4	10	1	0
9	10	16	Seroma	5	8	1	1
10	11	17	None	6	5	1	0
11	11	18	None	6	7	0	0
12	11	19	None	6	6	0	0
13	10	16	None	5	10	2	0

*MCL* Medial collateral ligament, *VAS* Visuel analog scale.

Donor-site complications occurred a few times but were transient. Paraesthesia along the saphenous territory was observed in three patients, which resolved after 3–4 months without long-term disability. Two patients developed seroma at the donor site, which required uncomplicated evacuation. One patient developed hematoma at the donor site, which resolved with conservative treatment. And one patient developed medial collateral ligament injury, which was repaired with anchor suture and healed.

## Discussion

Bone flaps or periosteal flaps are good options in cases such as atrophic nonunion. It also can be thought the first line of treatment for atrophic nonunion. In the case of large bone defects, a fibular flap could be used^11^. However, if there is no significant bone loss, vascularized periosteal flaps can be an ideal alternative^12^. The MFC periosteal flap presents crucial advantages, notably, its vascularity supporting bone healing, applicability to small bone defects, and its promotion of fracture healing through enhanced blood supply to the fracture line. Beyond bolstering local biology via increased blood flow, an additional benefit lies in the flap's capacity to generate bone tissue, further supporting the union. In the light of our study, we have achieved satisfactory outcomes with the pedicled periostal flap in the treatment of recalcitrant distal femoral nonunions. The attainment of these favorable outcomes is associated with establishing a well-vascularized environment—an essential component for optimal fracture healing with the MFC flap. The most important disadvantage of this method is that it cannot be used for the proximal 1/3 of the femur as a pedicled flap. The most important trick in placing the flap on the fracture line is decorticating the fracture site and fixing the flap there. Other important points are that the pedicle should not be stretched and rotated.

Various approaches have been described in the literature for the treatment of femoral nonunions. While biological stimulation methods (bone marrow injection, BMP7, PRP, LIPUS, and ESWT)^13–15^ can be applied conservatively, exchange nailing, dynamization of the nail, external fixation over the existing nail, plate augmentation, implantation of conventional iliac crest bone graft with the nail being left in place, and more complex management, such as bone flap, could be the methods for total restoration^15,16^. Fixation and cancellous bone grafting from the iliac crest is an effective method and should be considered as the first-line treatment of nonunions without bone defects. However, with some patients, the union sites are severely scarred and locally ischemic due to previous operations. For this reason, strong osteogenic activity is desirable apart from stable fixation. In this scenario, the achievement of bone union with conventional iliac bone grafting could be a formidable challenge. Finley et al. revealed that periosteal flap could produce bone^17^. The cambium layer resides at the lowest part of the periosteum close to the bone. This layer contained osteogenic progenitor cells which can be differentiated into osteoblast and new bone formation. Moreover, thin periosteal flaps were elastic and could be shaped readily according to any configuration in the recipient site. Therefore, they are good indications for nonunions with no significant bone defects and long-lasting (9–56 months) long-bone union^18^.

In their cadaver study, Yoshida et al.^16^ found that the pedicle bone graft could reach an average of 17.3 cm from the knee joint and at an average transposition ratio of 0.60. In our study, the mean distance between the origin of the DGA from the femoral artery and the periosteal flap was 10 cm. The mean transposition ratio was 16 ± 2.3. According to these results the periosteal MFC flap is sufficient for nonunions involving distal two-thirds of the femur.

Choudry et al.^19^ reported the treatment results of pedicled MFC periosteal bone flap in 3 of 11 patients with persistent nonunion of the femur. Yoshida and colleagues used pedicled MFC periosteal flaps in the treatment of femoral nonunion in two patients^16^. Hamada et al.^20^ modified the femoral periosteal bone flap in the lower and upper limb nonunion with conventional bone grafts in which pedicled femoral periosteal bone flaps were used in 3 of 21 patients for femoral nonunion. Guzzini and colleagues^21^ published a case report regarding femoral nonunion treated with pedicled MFC corticoperiosteal flap.

Vegas and colleagues^22^ reported that both methods were effective for the treatment of patients with small bone defects in their study that compared periosteal and corticoperiosteal MFC flaps. Periosteal flaps were able to completely cover the nonunion site, whereas the corticoperiosteal flaps did not; however, this did not cause adverse effects on the bone union. Hamada et al.^20^ compared periosteal bone flap with conventional iliac crest bone grafting in long-bone nonunion. According to their results, the average union time following periosteal flap was 2 months, compared with 5.5 months (*p* < 0.01), following iliac crest bone grafting. Choudry et al.^19^ achieved union at an average of 6.6 months in three patients, whereas Yoshida and colleagues^16^ achieved bone union in 7 months in one patient and 4 months in another one. In the study by Guzzini et al.^21^, the union was achieved after the femur nonunion operation with a pedicled periosteal flap in 3.5 months. The mean union time in our study was 5 ± 1 months.

The study by Aslantürk et al.^23^ focused on treating femoral nonunion using the sandwich technique with a strut allograft, achieving union in all 21 patients within 6.2 months. Despite the authors utilizing strut allografts in atrophic or oligotrophic nonunions, which do not inherently boost blood flow, satisfactory results and complete bone union were reported in all cases, with only one instance of superficial infection. However, only two patients in the study underwent multiple nonunion surgeries, distinguishing it from our research. In addition, the utilization of strut grafts in the mentioned study incurred a possible increase in treatment costs^23^. A notable drawback of our study, in comparison to the mentioned research, is that flap dissection involves a relatively more challenging technique and requires a learning curve.

Notwithstanding the favorable outcomes of our present study, it does not exclude limitations. First, our study is based on a retrospective design with small sample size and without a comparison group; since these cases are uncommon, gathering a large number of cases in a series is challenging. However, these limitations were mitigated by a long follow-up period, which was longer than the previously published studies.

In conclusion, the pedicled MFC flap for femur distal two-thirds has been effective in the treatment of recalcitrant femur nonunions without significant bone defect. There is no dependence on microvascular anastomosis. Besides, the operation time is relatively short, and the donor site morbidity is minimal. Surgeons may consider this flap as a useful reconstruction option for patients with previous failed femur nonunion treatments.

## Data Availability

The datasets generated during and/or analysed during the current study are available from the corresponding author on reasonable request.
